# Protocol: a highly sensitive RT-PCR method for detection and quantification of microRNAs

**DOI:** 10.1186/1746-4811-3-12

**Published:** 2007-10-12

**Authors:** Erika Varkonyi-Gasic, Rongmei Wu, Marion Wood, Eric F Walton, Roger P Hellens

**Affiliations:** 1 HortResearch, Mt Albert Research Centre, Private Bag 92169, Auckland, New Zealand

## Abstract

MicroRNAs (miRNAs) are a class of small non-coding RNAs with a critical role in development and environmental responses. Efficient and reliable detection of miRNAs is an essential step towards understanding their roles in specific cells and tissues. However, gel-based assays currently used to detect miRNAs are very limited in terms of throughput, sensitivity and specificity. Here we provide protocols for detection and quantification of miRNAs by RT-PCR. We describe an end-point and real-time looped RT-PCR procedure and demonstrate detection of miRNAs from as little as 20 pg of plant tissue total RNA and from total RNA isolated from as little as 0.1 μl of phloem sap. In addition, we have developed an alternative real-time PCR assay that can further improve specificity when detecting low abundant miRNAs. Using this assay, we have demonstrated that miRNAs are differentially expressed in the phloem sap and the surrounding vascular tissue. This method enables fast, sensitive and specific miRNA expression profiling and is suitable for facilitation of high-throughput detection and quantification of miRNA expression.

## Introduction

MicroRNAs (miRNAs) are families of short non-coding transcripts, arising from larger precursors with a characteristic hairpin secondary structure [reviewed in [1]]. Together with short interfering RNAs (siRNAs), miRNAs belong to a class of 19- to 25-nucleotide (nt) small RNAs that are essential for genome stability, development and differentiation, disease, cellular communication, signaling, and adaptive responses to biotic and abiotic stress [1-4]. A large proportion of miRNAs are highly conserved among distantly related species, from worms to mammals in the animal kingdom [1], and mosses to high flowering eudicots in plants [5,6].

Currently, over 4000 miRNA sequences from vertebrates, flies, worms, plants and viruses are annotated in the Sanger Centre miRBase Database [version 9.0, October 2006; [7]]. In animals, miRNAs appear predominantly to inhibit translation by targeting partially complementary sequences located within the 3' untranslated region (UTR) of mRNA [reviewed in [8]]. The majority of animal miRNAs appear to be operating at several levels, regulating multiple targets implicated in various molecular functions and biological processes [1]. In plants, miRNAs repress gene expression by acting either on near-perfect complementary sequences in mRNA coding region to guide cleavage and translational repression [9-12], or in at least one example, on DNA to guide chromatin remodelling [13]. The majority of plant miRNA targets are developmentally important transcription factors [14,15] and stress-regulated genes [16,17]. Thus, ectopic expression of miRNAs [9,10,13,18-20] or misexpression of miRNA-resistant target mRNAs can induce strong developmental phenotypes [reviewed in [21]]. It has been proposed that plant miRNAs act mainly by clearing of the mRNA of the target regulatory genes during the cell-fate changes [15,22,23]. There is also evidence for quantitative action of plant miRNAs in quenching the target gene activity rather than eliminating it completely [24,25]. Furthermore, several miRNAs were detected in the phloem sap, suggesting a long-distance signaling role [26], in contrast to several miRNAs with demonstrated cell-autonomous expression and effects [27,28].

This complexity in miRNA modes of action demonstrates that reliable detection and quantification of miRNA expression in specific tissues is essential for better understanding of miRNA-mediated gene regulation. Although miRNA represent a relatively abundant class of transcripts, their expression levels vary greatly among different cells and tissues. Conventional technologies such as cloning, northern hybridization and microarray analysis are widely used but may not be sensitive enough to detect less abundant miRNAs. Furthermore, intensive small RNA sequencing revealed a very complex small RNA population in plants. Unlike mammals, which have relatively simple small RNA populations comprising mainly miRNAs and no siRNAs [29], plants have a hugely complex small RNA fraction. It is comprised of both miRNAs and endogenous siRNAs derived from repetitive sequences, intergenic regions and genes [14,30]. This complexity renders miRNAs highly under-represented in the small RNA fraction and further affects detection methods such as cloning and microarray hybridization.

Poor sensitivity and low throughput of conventional technologies can be overcome by using a sensitive reverse transcription-polymerase chain reaction (RT-PCR) detection method. However, because of their small size, detection of miRNAs by PCR is technically demanding. A number of specific quantitative RT-PCR (qRT-PCR) techniques were developed and optimised for miRNA detection, including real-time methods based upon reverse transcription (RT) reaction with a stem-loop primer followed by a TaqMan PCR analysis [31,32]. The stem-loop reverse transcription primers provide better specificity and sensitivity than linear primers because of base stacking and spatial constraint of the stem-loop structure [31]. Detection sensitivity is further increased by a pulsed RT reaction [32]. However, these methods were optimized for detection of mammalian miRNAs and require individual miRNA-specific fluorescent probes, making them very costly to many laboratories and not amenable for high-throughput analysis of a large number of miRNAs. Alternatively, qRT-PCR miRNA detection kits and primer sets are commercially available, but are very costly and thus not suitable for high-throughput miRNA analysis. Indeed, the available plant miRNA expression data are mainly results of gel-based assays.

Here we describe and provide protocols for an end-point and real-time looped RT-PCR procedure. We demonstrate detection of miRNAs from as little as 20 pg of plant tissue total RNA and from total RNA isolated from as little as 0.1 μl of phloem sap.

**Figure 1:**
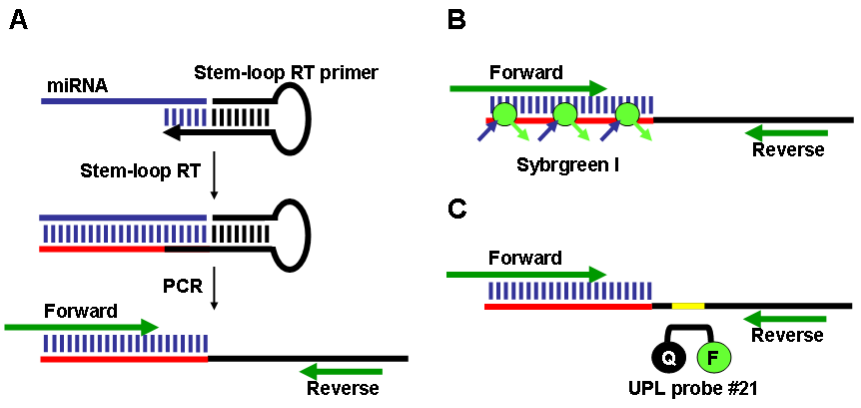
**Schematic showing stem-loop RT-PCR miRNA assays**. A. Stem-loop RT followed by end-point PCR. Stem-loop RT primers bind to the 3' portion of miRNA molecules, initiating reverse transcription of the miRNA. Then, the RT product is amplified using a miRNA specific forward primer and the universal reverse primer. B. SYBR Green I assay. C. Universal ProbeLibrary (UPL) probe assay. Highlighted in yellow is the UPL probe #21 binding site.

The expression of a miRNA was detected using a two-step process. First, the stem-loop RT primer designed according to Chen et al. [31] was hybridized to the miRNA molecule, and then reverse transcribed in a pulsed RT reaction. Next, the RT product was amplified using a miRNA-specific forward primer and the universal reverse primer (Figure 1A). The amplification product was visualized on an agarose gel by ethidium bromide staining. The amplification was also performed in real-time, using the SYBR Green I assay (Figure 1B). In addition, we have developed a Universal ProbeLibrary (UPL; Roche Diagnostics) RT-PCR method for miRNA detection and quantification that provides increased specificity when analysing expression of low abundant miRNAs (Figure 1C).

## Materials

### Plant material and phloem sap collection

*Arabidopsis thaliana *Columbia, *Cucurbita maxima *'Crown' (pumpkin) and *Cucumis sativus *'Telegraf' (cucumber) plants were grown in a greenhouse under natural daylight conditions. Phloem sap was collected from well-watered plants, as previously described [26].

### miRNA sequences, primers and probes

Plant miRNA genes were selected from the Sanger Institute miRBase Sequence Database [33]. UPL probe #21 was obtained from universal probe library database (Roche Diagnostics). Stem-loop RT primers were designed according to Chen et al. [31]. Sequence data are presented in Table 1.

**Table 1:** miRNA, primer and probe sequences

miR156	miRNA sequence	UGACAGAAGAGAGUGAGCAC
	Antisense probe	GTGCTCACTCTCTTCTGTCA
	Sense probe	TGACAGAAGAGAGTGAGCAC
	RT primer	GTCGTATCCAGTGCAGGGTCCGAGGTATTCGCACTGGATACGACGTGCTC
	Forward primer	GCGGCGGTGACAGAAGAGAGT
	UPL RT primer	GTTGGCTCTGGTGCAGGGTCCGAGGTATTCGCAC**CAGAGCCA**ACGTGCTC
miR156RC	RT primer	GTCGTATCCAGTGCAGGGTCCGAGGTATTCGCACTGGATACGACTGACAG
	Forward primer	GCGGCGTGTGCTCACTCTCT

miR159	miRNA sequence	UUUGGAUUGAAGGGAGCUCUA
	Antisense probe	TAGAGCTCCCTTCAATCCAAA
	Sense probe	TTTGGATTGAAGGGAGCTCTA
	RT primer	GTCGTATCCAGTGCAGGGTCCGAGGTATTCGCACTGGATACGACTAGAGC
	Forward primer	CGGCGGTTTGGATTGAAGGGA
	UPL RT primer	GTTGGCTCTGGTGCAGGGTCCGAGGTATTCGCAC**CAGAGCCA**ACTAGAGC

miR159RC	RT primer	GTCGTATCCAGTGCAGGGTCCGAGGTATTCGCACTGGATACGACTTTGGA
	Forward primer	CGGCGGTAGAGCTCCCTTCAA

miR167	miRNA sequence	UGAAGCUGCCAGCAUGAUCUA
	Antisense probe	TAGATCATGCTGGCAGCTTCA
	Sense probe	TGAAGCTGCCAGCATGATCTA
	RT primer	GTCGTATCCAGTGCAGGGTCCGAGGTATTCGCACTGGATACGACTAGATC
	Forward primer	TCGCGTGAAGCTGCCAGCAT
	UPL RT primer	GTTGGCTCTGGTGCAGGGTCCGAGGTATTCGCAC**CAGAGCCA**ACTAGATC

miR167RC	RT primer	GTCGTATCCAGTGCAGGGTCCGAGGTATTCGCACTGGATACGACTGAAGC
	Forward primer	GGCGGTAGATCATGCTGGCA

miR171	miRNA sequence	UGAUUGAGCCGCGCCAAUAUC
	Antisense probe	GATATTGGCGCGGCTCAATCA
	RT primer	GTCGTATCCAGTGCAGGGTCCGAGGTATTCGCACTGGATACGACGATATT
	Forward primer	TTCCTTGATTGAGCCGCGCC
	UPL RT primer	GTTGGCTCTGGTGCAGGGTCCGAGGTATTCGCAC**CAGAGCCA**ACGATATT

miR166	miRNA sequence	UCGGACCAGGCUUCAUUCCCC
	RT primer	GTCGTATCCAGTGCAGGGTCCGAGGTATTCGCACTGGATACGACGGGGAA
	Forward primer	TCGCTTCGGACCAGGCTTCA
	UPL RT primer	GTTGGCTCTGGTGCAGGGTCCGAGGTATTCGCAC**CAGAGCCA**ACGGGGAA

Universal	Reverse primer	GTGCAGGGTCCGAGGT

*CmRBS*	Forward primer	ATGGCTTCCATCGTCTCATCCGCC
	Reverse primer	TTGTCGAAGCCAATGACTCTGATGAA

*CmPP16*	Forward primer	GTGGTAAAGGACTTCAAGCCCACGACC
	Reverse primer	ATGGGTTTGAAGAAGCCAAGCCACTTA

UPL probe #21 reverse complement sequence is highlighted in bold.

**NOTE: ***The specificity of stem-loop RT primers to individual miRNA is conferred by a six nucleotide extension at the 3' end; this extension is a reverse complement of the last six nucleotides at the 3' end of the miRNA (*Figure 1A*). Forward primers are specific to the miRNA sequence but exclude the last six nucleotides at the 3' end of the miRNA. A 5' extension of 5–7 nucleotides is added to each forward primer to increase the melting temperature; these sequences were chosen randomly and are relatively GC-rich. We used standard primer design software to assess the quality of forward primers*.

## Protocols

### Equipment

Standard laboratory equipment including a thermal cycler is required for pulsed reverse transcription and end-point PCRs. A real-time thermal cycler is required for SYBR Green I and UPL probe assays. All our reverse transcription reactions and end-point PCR analyses were performed on Mastercycler (Eppendorf, Hamburg, Germany). All real-time PCR analyses were performed on LightCycler 1.5 (Roche Diagnostics, Mannheim, Germany).

### RNA template

RNA should be handled according to standard laboratory practices to avoid RNase contamination. Avoid RNA purification methods that use RNA-binding glass-fiber filters that do not quantitatively recover small RNA species. In our hands, both non-denatured RNA and RNA denatured by incubation at 65°C for 5 minutes produced similar results.

**NOTE: ***Total RNA used in our experiments was isolated using the TRIzol reagent (Invitrogen, Carlsbad, CA). High molecular weight (HMW)RNA was purified with RNAqueous kit (Ambion, Austin, TX). Low molecular weight (LMW)RNA present in the flow-through was precipitated as described previously *[26]*. The concentration of RNA was determined using the NanoDrop ND-1000 Spectrophotometer (NanoDrop Technologies, Wilmington, DE)*.

*For RNA gel blot analyses, HMW RNA was separated on a formaldehyde-containing 1% agarose gel and transferred overnight to a Hybond-N+ membrane (GE Healthcare, formerly Amersham Biosciences, Buckinghamshire, UK). LMW RNA was separated on a 7 M urea/15% polyacrylamide gel and transferred overnight to a Hybond-XL membrane (GE Healthcare). The membranes were UV cross-linked and prehybridized at 40–45°C for 1 h in hybridization buffer (0.5 M Na*_2_*HPO*_4_*, 1 mM EDTA, 1% BSA, and 7% SDS). DNA oligo probes were end labeled by the forward reaction using 10 units of T4 polynucleotide kinase (Invitrogen) with the supplied buffer, to which was added 300 nM [γ-*^32^*P]ATP (3000 Ci/mmol) for 10 min at 37°C. Unincorporated *^32^*P-label was removed using ProbeQuant G-50 microcolumns (GE Healthcare). Probes were denatured at 94°C for 5 min, added to the hybridisation buffer and hybridisation was allowed to proceed at 40°C overnight. The membrane was then washed twice, 15 min each in 2× SSC, 0.1% SDS at 50°C. Hybridization signal was detected using a Typhoon scanner (GE Healthcare)*.

### Stem-loop pulsed reverse transcription protocol

1. Prepare an RT master mix by scaling the volumes listed below to the desired number of RT reactions. If testing many RNA samples for one miRNA, prepare a 'no RNA' master mix; if testing for many different miRNAs in one sample, prepare a 'no RT primer' master mix. Include 10% overage to cover pipetting errors. Also prepare the minus RT controls by omitting reverse transcriptase from the reactions and water controls by adding nuclease-free water in place of RNA. Keep on ice and work in the cold room if handling large number of samples.

• For a 'no RNA' master mix, add the following components to a nuclease-free microcentrifuge tube:

0.5 μl 10 mM dNTP mix

11.15 μl nuclease-free water

1 μl of appropriate stem-loop RT primer (1 μM)

• For a 'no RT primer' master mix, add the following components to a nuclease-free microcentrifuge tube:

0.5 μl 10 mM dNTP mix

11.15 μl nuclease-free water

1 μl of appropriate RNA template

• Heat mixture to 65°C for 5 minutes and incubate on ice for 2 minutes.

• Centrifuge briefly to bring solution to the bottom of the tube and add:

4 μl 5× First-Strand buffer

2 μl 0.1 M DTT

0.1 μl RNaseOUT (40 units/μl)

0.25 μl SuperScript III RT (200 units/μl)

• Mix gently and centrifuge to bring solution to the bottom of the tube.

2. Assemble the RT reaction.

• Aliquot the appropriate amount of the RT master mix (19 μl).

• Add 1 μl RNA template to 'no RNA' master mix or

• Add 1 μl of appropriate stem-loop RT primer (1 μM) to 'no RT primer' master mix

• Mix gently and centrifuge to bring solution to the bottom of the tube.

3. Perform pulsed RT:

• Load thermal cycler and incubate for 30 min at 16°C, followed by pulsed RT of 60 cycles at 30°C for 30 s, 42°C for 30 s and 50°C for 1 s.

• Incubate at 85°C for 5 min to inactivate the reverse transcriptase.

**NOTE: ***RT reaction volume can be scaled down to 10 μl*.

### End-point PCR protocol

1. Prepare a PCR master mix by scaling the volumes listed below to the desired number of amplification reactions. Include 10% overage to cover pipetting errors. Also prepare water controls by adding nuclease-free water in place of the RT product.

• Add the following components to a nuclease-free microcentrifuge tube:

15.4 μl nuclease-free water

2 μl 10× PCR buffer

0.4 μl 10 mM dNTP mix

0.4 μl forward primer (10 μM)

0.4 μl reverse primer (10 μM)

0.4 μl Advantage 2 Polymerase mix

• Mix gently and centrifuge to bring solution to the bottom of the tube.

2. Aliquot 19 μl of the PCR master mix into PCR tubes and add 1 μl RT product.

3. Place reactions in a preheated (94°C) thermal cycler heat block and incubate at 94°C for 2 min, followed by 20–40 cycles of 94°C for 15 s and 60°C for 1 min.

4. Analyse reaction products by electrophoresis on a 4% agarose gel in 1× TAE.

**NOTE: ***We tested several commercial thermostable DNA polymerases. In our hands, the most consistent results were obtained using Advantage 2 PCR Polymerase Mix (Clontech, Mountain View, CA)*.

### miRNA SYBR Green I assay protocol

1. Prepare 5× LightCycler FastStart SYBR Green I master mix (Roche Diagnostics) according to manufacturer's instructions.

2. Prepare a PCR master mix by scaling the volumes listed below to the desired number of amplification reactions. Include 10% overage to cover pipetting errors.

• Add the following components to a nuclease-free microcentrifuge tube:

12 μl nuclease-free water

4 μl SYBR Green I master mix

1 μl forward primer (10 μM)

1 μl reverse primer (10 μM)

• Mix gently and centrifuge to bring solution to the bottom of the tube.

• Store in cooling block or on ice.

3. Perform real-time PCR:

• Place required number of LightCycler capillaries in precooled centrifuge adapters.

• Pipet 18 μl master mix into each LightCycler capillary.

• Add 2 μl RT product.

• Seal each capillary with a stopper.

• Place capillaries into the LightCycler carousel and spin in the carousel centrifuge.

• Incubate samples at 95°C for 5 min, followed by 35–45 cycles of 95°C for 5 s, 60°C for 10 s, and 72°C for 1 s.

• For melting curve analysis, denature samples at 95°C, then cool to 65°C at 20°C per second. Collect fluorescence signals at 530 nm wavelength continuously from 65°C to 95°C at 0.2°C per second.

4. Analyse results using the LightCycler software.

• Prepare standard curves for each primer set by using dilution series of the experimental sample expected to have the highest expression. Use at least 3 points (or one point per log of concentration whichever is greater).

• Alternatively, standard curves can be prepared from dilution series of an appropriate RNA oligonucleotide, e.g. when detecting artificial miRNAs or siRNAs.

• Perform a Relative Quantification-Monocolor Analysis.

**NOTE: ***PCR reaction volume can be scaled down to 10 μl. Perform PCR in at least three replicates*.

### miRNA UPL probe assay protocol

1. Prepare 5× LightCycler TaqMan master mix (Roche Diagnostics) according to manufacturer's instructions.

2. Prepare a PCR master mix by scaling the volumes listed below to the desired number of amplification reactions. Include 10% overage to cover pipetting errors.

• Add the following components to a nuclease-free microcentrifuge tube:

11.8 μl nuclease-free water

4 μl TaqMan master mix

1 μl forward primer (10 μM)

1 μl reverse primer (10 μM)

0.2 μl UPL probe #21 (10 μM)

• Mix gently and centrifuge to bring solution to the bottom of the tube.

• Store in cooling block or on ice.

3. Perform real-time PCR as described for miRNA SYBR Green I assay (omit melting curve analysis).

4. Analyse results using the LightCycler software as described above.

## Comments

### The sensitivity of stem-loop RT-PCR assays

**Figure 2:**
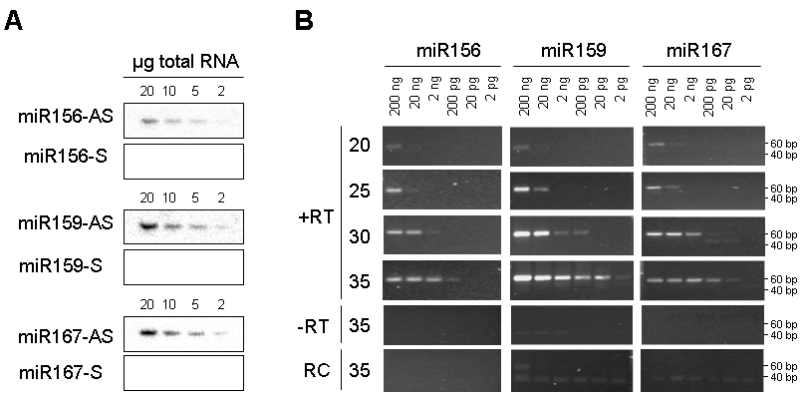
**The sensitivity of the stem-loop RT-PCR assay**. A. Gel blot analyses of miR156, miR159 and miR167 expression. AS, antisense probe; S, sense probe. B. Stem-loop RT-PCR analyses of miR156, miR159 and miR167 expression. The amounts of RNA used for reverse transcription reactions are indicated on the top. PCR cycle numbers are indicated on the left.

Gel blot analyses of *Arabidopsis *seedling shoot RNA dilution series established that the abundant plant miRNAs (miR156, miR159 and miR167) can be detected from as little as 2 μg of total RNA (Figure 2A). To establish the sensitivity of the stem-loop RT-PCR, further step-wise dilutions of total RNA obtained from *Arabidopsis *seedling shoots were prepared. This amplification was performed in a semi-quantitative manner, using 20–35 cycles (Figure 2B). This analysis showed that miR156, miR159 and miR167 can be detected from as little as 20 pg total RNA after 35 cycles of PCR. At this number of cycles, no amplification or a weak band of primer-dimers was obtained in minus-RT control and in stem-loop RT-PCR reactions with primers designed to amplify the reverse complement (RC) strand of miRNA. Similarly, no amplification was obtained from the water control (data not shown). However, 40 or more cycles of PCR often gave rise to some non-specific amplification in control reactions (data not shown). Therefore, all further end-point amplification reactions were limited to 35 cycles.

### The specificity of stem-loop RT-PCR assays

**Figure 3:**
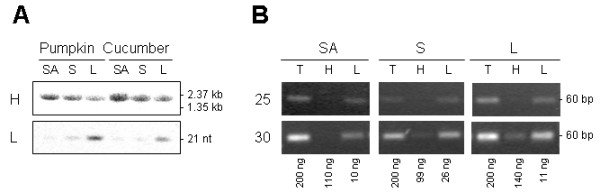
**The specificity of the stem-loop RT-PCR assay**. A. Gel blot analysis of pri- and mature miR156 expression. High molecular weight (H) and low molecular weight (L) RNA were purified from 20 μg of total RNA, separated by electrophoresis, transferred and hybridised with miR156 antisense probe. B. Stem-loop RT-PCR analyses of miR156 expression. 200 ng total RNA (T), high molecular weight RNA purified from 200 ng total RNA (H) and low molecular weight RNA purified from 200 ng total RNA (L) were subjected to stem-loop RT PCR. The amount of input RNA as measured by NanoDrop is indicated below. PCR cycle numbers are indicated to the left. SA, shoot apex; S, stem: L, leaf.

PCR amplification methods can lack specificity for mature miRNAs. Mature miRNAs are processed from large capped and polyadenylated transcripts (primary or pri-miRNA) that first give rise to short-lived hairpin intermediates (pre-miRNA) and finally to mature single stranded miRNAs. In some instances, total RNA contains large amounts of pri-miRNAs, as established for pri-miR156 in pumpkin and cucumber tissues (Figure 3A). RNA gel blot analyses clearly show higher expression of pri-miR156 in the shoot tip and stem than in leaf tissue, and higher expression of mature miRNA in leaf than in shoot tip and stem. To investigate the ability of stem-loop RT-PCR assays to differentiate between mature miRNAs and their pri-miRNAs, the reactions were performed using pumpkin tissue total RNA and the high molecular weight (HMW) and low molecular weight (LMW) RNA purified from total RNA. Similar amounts of amplification product from total and LMW RNA were obtained after 25 and 30 cycles (Figure 3B). The slightly more efficient amplification of total RNA than LMW RNA was the result of RNA losses during purification, as only 60–75% RNA was recovered. Some amplification visible in the HMW RNA fraction is more likely to be the result of contamination of the HMW RNA with LMW RNA, rather than amplification of the primary miRNA, as there is less amplification in the shoot tip HMW RNA than in leaf HMW RNA. Similar results were obtained with cucumber tissue RNA (data not shown). These results suggest that the stem-loop RT-PCR assay is highly specific for mature miRNAs.

### Detection of miRNAs from small amounts of tissue and phloem sap RNA

**Figure 4:**
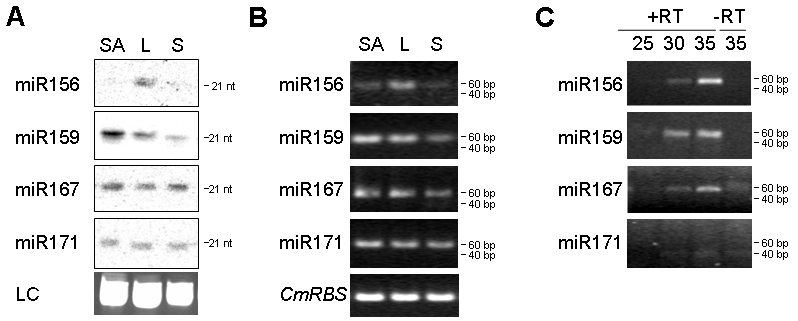
**Detection of miRNAs in tissue and phloem sap**. A. Gel blot analyses of miRNA expression in pumpkin shoot apex (SA), leaf (L) and stem (S). An ethidium bromide-stained prominent band of tRNA was used as the loading control (LC). B. Stem-loop end-point RT-PCR analyses of miRNA expression. miRNAs were amplified using 28 cycles of PCR. Pumpkin *RUBISCO *(*CmRBS*) mRNA was amplified using 30 cycles of standard PCR. C. Stem-loop end-point RT-PCR analyses of miRNA expression in pumpkin phloem sap. The number of PCR cycles is indicated on the top. miR156, miR159 and miR167, but not miR171 were detected.

We further analysed the expression of several miRNAs in pumpkin tissues. RNA gel blot analysis suggested higher expression in leaf than in the shoot apex or stem tissue for miR156; highest expression in the shoot apex, followed by leaf and low expression in the stem tissue for miR159; miR167 and miR171 showed similar levels of expression across all analysed tissues (Figure 4A). Comparable results were obtained using 28 cycles of stem-loop RT-PCR (Figure 4B).

To test the ability of the stem-loop RT-PCR assay to detect miRNA sequences in very small amounts of rare biological samples, pumpkin and cucumber phloem sap RNA were used. The RNA concentration in pumpkin and cucumber phloem sap is in the range of 300–400 ng/ml [26]. Phloem sap is enriched for some miRNAs and siRNAs. miR156, miR159 and miR167 were cloned from the sap RNA and were detectable in RNA derived from 1 ml of the sap by RNA gel blot analysis [26]. The stem-loop RT-PCR identified miR156, miR159 and miR167 in RNA purified from as little as 0.1 μl phloem sap (Figure 4C). miR171, previously shown not to be phloem-mobile [26,27], could not be detected in the phloem sap RNA by this assay.

### Real-time detection of miRNAs using the SYBR Green I assay and UPL probe assay

**Figure 5:**
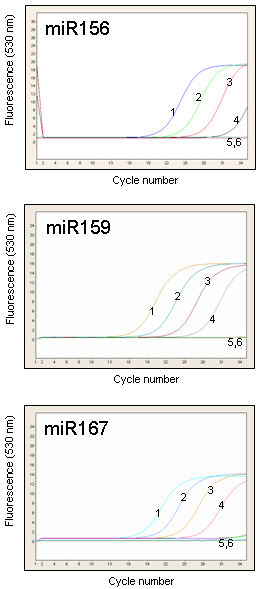
**miRNA SYBR Green I assay**. Real-time PCR amplification profiles of miR156, miR159 and miR167. The amounts of RNA used for stem-loop pulsed RT were as follows: 1, 20 ng; 2, 2 ng; 3, 200 pg; 4, 20 pg; 5, 20 ng minus-RT control; 6, water control.

An aliquot of cDNA previously used to establish the sensitivity of end-point PCRs was amplified in real-time, using 35 cycles of the SYBR Green I assay (Figure 5). We were again able to detect miR156, miR159 and miR167 from as little as 20 pg total RNA without significant amplification in minus-RT control and the water control. However, a larger number of amplification cycles often resulted in non-specific amplification in the 40–80 bp range that was difficult to distinguish from the desired amplification product. These non-specific products were found in the minus-RT and water controls and were often indistinguishable from the specific PCR products by melting-curve analysis.

Dual labeled hydrolysis probes such as TaqMan (Applied Biosystems) and more recently UPL (Roche Diagnostics) are routinely used to increase specificity of real-time quantitative PCR (qPCR) assays. TaqMan probes have been used successfully to detect mammalian miRNAs [31,32]. However, a unique TaqMan probe is required for each miRNA sequence, which may be impractical when screening large numbers of miRNAs. To investigate the efficiency of a single, universal hybridization probe, we developed a miRNA UPL probe assay that utilises a short hydrolysis probe of 8 nucleotides, of which one is a locked nucleic acid (LNA) to increase binding specificity and melting temperature. The stem-loop oligonucleotides were redesigned to include a UPL probe #21 (Roche Diagnostics) reverse complement sequence in the stem region between the miRNA-specific sequence and the universal reverse oligonucleotide sequence (Figure 1C). Pulsed stem-loop RT reactions were performed on an RNA dilution series, followed by UPL qPCR.

**Figure 6:**
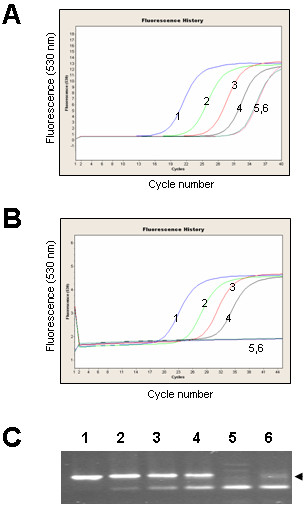
**miRNA UPL probe assay**. A. SYBR Green I assay for miR166. Negative control reactions (minus-RT and water) produced detectable amplicons after 40 PCR cycles. B. UPL probe assay for miR166. No fluorescence was detected in the negative control reactions after 45 cycles of PCR. C. UPL probe assay amplification products for miR166 separated by gel electrophoresis on 4% agarose showing specific and non-specific amplification bands obtained after 45 cycles of PCR. Arrowhead indicates the expected size of amplicons. 1, 20 ng RNA; 2, 2 ng RNA; 3, 200 pg RNA; 4, 20 pg RNA; 5, 20 ng RNA minus-RT control; 6, water control.

We compared miR166 amplification curves obtained using SYBR Green I assay and the UPL probe assay. At 40 cycles of SYBR Green I assay amplification we could detect non-specific products in the minus-RT and water controls (Figure 6A). Melting-curve analysis could not distinguish between the specific and non-specific PCR products, but cloning and sequencing of amplicons derived from minus-RT control revealed that they were concatenated primer sequences (data not shown).

At 45 cycles of UPL probe assay amplification, amplification curves correlated with the concentration of the RNA (Figure 6B). Neither of the negative controls (minus-RT and water) gave a detectable signal, though non-specific amplification bands from minus-RT control and water could be seen on the agarose gels (Figure 6C). Cloning and sequencing confirmed that plus-RT reaction products were the expected amplicons. Sequencing of the minus-RT control products revealed that they were concatenated primer sequences.

**NOTE: ***The concept of stem-loop RT-PCRs has been used in multiplex assays. We have not tested multiplexing with the UPL probe assay*.

### Quantification of miRNA expression in vascular tissue and phloem sap

Finally, we used the miRNA UPL assay to quantify miRNA expression levels in pumpkin vascular tissue and the phloem sap. miRNA expression data were normalized to pumpkin phloem RNA binding protein, *CmPP16*, as this mRNA species was earlier shown to be expressed both in the vascular tissue and in the phloem sap [34].

**Figure 7:**
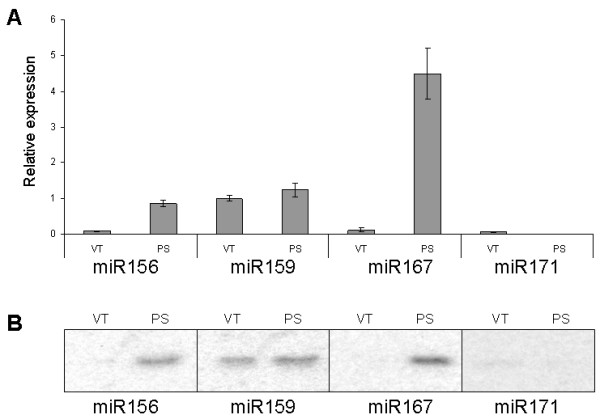
**Expression of miRNAs in vascular tissue and phloem sap**. A. Expression of miRNAs in pumpkin vascular tissue (VT) and phloem sap (PS) detected by miRNA UPL assay. Samples containing 10 ng total RNA isolated from pumpkin vascular bundles and phloem sap each were subjected to stem-loop RT reactions and subsequent UPL qPCR. The PCR was performed in three replicates and miRNA expression was normalized against *CmPP16 *and expressed as a ratio with vascular tissue miR159 expression, which was set arbitrarily at 1. B. Expression of miRNAs detected by gel blot analyses.

This assay established that the phloem sap is enriched for miR156 and miR167; phloem sap showed a 10-fold increase in miR156 expression and a 20-fold increase in miR167 expression compared with the surrounding vasculature. The abundance of miRNA159 was similar in the phloem sap and in the surrounding vascular tissue. Only relatively low levels of miR171 expression were detected in the vascular tissue, but very little or no expression was detectable in the phloem sap (greater than 10-fold below the levels detected in the vasculature; Figure 7A). The results were comparable to those obtained by RNA gel blot analyses (Figure 7B) and spatial analysis using *in situ *hybridization [35] that revealed high expression of several miRNA, including miR159, but not miR167, in the vascular bundles of *Arabidopsis thaliana *and *Nicotiana benthamiana*.

## Conclusion

The stem-loop pulsed RT-PCR assays described here are rapid, sensitive, specific and convenient for screening a large number of miRNAs quickly and inexpensively. They require considerably less tissue and time compared with standard gel-based methods. We have demonstrated their reliability, sensitivity and specificity using plant tissue RNA and phloem sap RNA. These assays enable miRNA expression profiling from as little as 20 pg RNA and as little as 0.1 μl phloem sap.

It is envisaged that this approach will have application in detection and quantification of miRNAs across kingdoms, as well as for other small RNA sequences such as artificial miRNAs and short interfering RNAs (siRNAs).

## Competing interests

The author(s) declare that they have no competing interests.

## Authors' contributions

EV-G conceived of the project, designed the experiments, conducted the gel-blot, end-point and real-time PCR analyses and prepared the manuscript. RW carried out the real-time PCR, cloning and sequencing and prepared the manuscript. MW conceived of the UPL probe assay. EFW acquired the funding and contributed to and edited the manuscript. All authors read and approved the final manuscript. RPH has given final approval of the version to be published.
